# Antibody gene features associated with binding and functional activity in malaria vaccine-derived human mAbs

**DOI:** 10.1038/s41541-024-00929-6

**Published:** 2024-08-10

**Authors:** Camila H. Coelho, Susanna Marquez, Bergeline C. Nguemwo Tentokam, Anne D. Berhe, Kazutoyo Miura, Vishal N. Rao, Carole A. Long, Ogobara K. Doumbo, Issaka Sagara, Sara Healy, Steven H. Kleinstein, Patrick E. Duffy

**Affiliations:** 1 Laboratory of Malaria Immunology and Vaccinology, National Institute of Allergy and Infectious Diseases, National Institutes of Health, Bethesda, MD 20892 USA; 2 Department of Microbiology, Icahn School of Medicine at Mount Sinai, New York, NY 10029 USA; 3 C-VARPP- Center for Vaccine Research and Pandemic Preparedness, Icahn School of Medicine at Mount Sinai, New York, NY 10029 USA; 4 Immunology Precision Institute, Icahn School of Medicine at Mount Sinai, New York, NY 10029 USA; 5 Department of Pathology, Yale School of Medicine, New Haven, CT 06520 USA; 6 Laboratory of Malaria and Vector Research, National Institute of Allergy and Infectious Diseases, NIH, Rockville, MD USA; 7 Graduate School of Biomedical Sciences, Icahn School of Medicine at Mount Sinai, New York, NY 10029 USA; 8 Malaria Research and Training Center, University of Sciences, Techniques and Technologies, Bamako, Mali; 9 Program in Computational Biology and Bioinformatics, Yale University, New Haven, CT 06511 USA; 10 Department of Immunobiology, Yale School of Medicine, New Haven, CT 06520 USA

**Keywords:** Malaria, Protein vaccines

## Abstract

The impact of adjuvants on malaria vaccine-induced antibody repertoire is poorly understood. Here, we characterize the impact of two adjuvants, Alhydrogel® and AS01, on antibody clonotype diversity, binding and function, post malaria vaccination. We expressed 132 recombinant anti-Pfs230D1 human monoclonal antibodies (mAbs) from participants immunized with malaria transmission-blocking vaccine Pfs230D1, formulated with either Alhydrogel® or AS01. Anti-Pfs230D1 mAbs generated by Alhydrogel® formulation showed higher binding frequency to Pfs230D1 compared to AS01 formulation, although the frequency of functional mAbs was similar between adjuvant groups. Overall, the AS01 formulation induced anti-Pfs230D1 functional antibodies from a broader array of germline sequences versus the Alhydrogel® formulation. All mAbs using IGHV1-69 gene from the Alhydrogel® cohort bound to recombinant Pfs230D1, but did not block parasite transmission to mosquitoes, similar to the IGHV1-69 mAbs isolated from the AS01 cohort. These findings may help inform vaccine design and adjuvant selection for immunization with *Plasmodium* antigens.

## Introduction

Despite intense efforts to eradicate malaria, the disease still affects hundreds of millions of people worldwide, of which over 90% live in Sub-Saharan Africa. Recent progress in malaria control has stalled, and even reversed in some areas^1^, highlighting the need for new prevention tools. Although two malaria vaccines have been licensed and recommended by WHO to prevent clinical malaria in children^2^, and others are in advanced stages of clinical trials^3^, long-term high-level protection has not yet been achieved. Many malaria vaccine candidates rely on antibody effector mechanisms and newer adjuvants, thus the antibody repertoire response to malaria vaccines including newer adjuvants require characterization to guide vaccine improvement.

Alhydrogel® is an adjuvant comprised of aluminum hydroxide gel, widely used in licensed vaccines for almost a century^4^. Alhydrogel® generally induces a Th2-type immune response, and is often considered the “benchmark” adjuvant in comparative human studies with new adjuvants^5^. However, novel adjuvants developed in recent decades have now been included in licensed vaccines^6,7^. AS01 is an adjuvant system that incorporates the TLR4 ligand, monophosphoryl lipid A, and a liposomal preparation of saponin fraction QS21^8^. AS01 efficiently promotes humoral and cellular immune responses, including CD4 T cells, that, in turn, further assist B cells in generating high-affinity antibodies. Recently, a study highlighted the ability of AS01 to enhance the hepatitis B surface antigen (HBsAg) antibody response versus aluminium-based adjuvants: De Mot et al. measured antibody-associated signatures within whole blood transcriptomes using linear regression and linked 300 genes to increased antibody response^9^.

Previous studies have shown that Alhydrogel® might not be efficient when used in influenza vaccine formulations^10,11^, whereas oil-in-water adjuvants, such as MF59 and AS03, induced higher antibody titers and neutralization rates against influenza viruses^12,13^. Additionally, several malaria vaccine candidates failed clinical trials when formulated with Alhydrogel®-based adjuvants^14,15^. Thus, improved vaccines are being developed and employ novel adjuvants, such as the recently approved pediatric RTS,S vaccine (Mosquirix®, GSK) that uses AS01E adjuvant similar to the AS01B used in the adult shingles vaccine Shingrix from GSK.

Conventional serological approaches used to characterize immune responses during malaria vaccination do not define the properties of the antibody genes elicited in response to vaccination. The genes that encode the variable regions of antibodies are called V (Variable) genes. These genes are part of a larger family of genes known as immunoglobulin (Ig) genes. V genes are highly diverse and exist in multiple copies within the genome. They provide the genetic blueprint for generating the diversity of antigen-binding sites found in antibodies. Here, we sought to characterize these properties by sorting antigen-specific memory B cells from healthy Malian adults immunized with Pfs230D1, a malaria transmission-blocking vaccine (TBV) candidate targeting the parasite gamete stage, formulated with either Alhydrogel® or AS01. Our results dissect human antibody responses at gene, binding and functional levels, after immunization with two different adjuvants, and can help inform malaria vaccine design and adjuvant selection for clinical studies aiming to elicit strong anti-malarial responses.

## Results

### Heavy chain V gene usage among binding and functional alhydrogel and AS01-derived Pfs230D1 mAbs

**Fig. 1:**
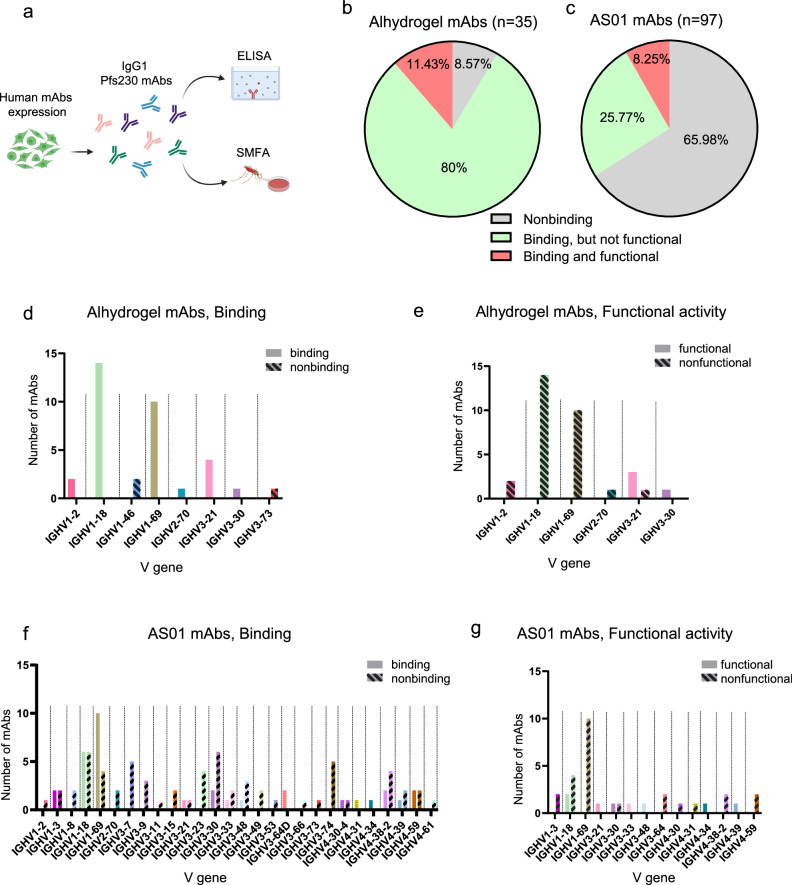
Binding and functional activity of Pfs230D1 mAbs and their relationship with heavy chain V gene usage. **a** Human IgG1 mAbs obtained from Pfs230D1-sorted single B cells were expressed in mammalian cells and tested for binding to Pfs230D1 (ELISA). Functional activity was determined as Transmission Reducing Activity (TRA), assessed by Standard Membrane Feeding Assay using NF54 strain *P. falciparum* parasites fed to *Anopheles* mosquitoes. Figure was created using Biorender.com. **b** Percentage of human mAbs obtained from Alhydrogel® and **c** AS01 vaccinees that bound to recombinant Pfs230D1 and were functional in vivo (TRA > 75% at 100 μg/mL). **d**–**g** Alhydrogel**®** and AS01 mAbs were grouped as **d**, **f** binding and non-binding and **e**, **g** functional and non-functional, and then are shown according to their V gene classification.

To assess the relationship between V gene usage and antibody binding or functional activity, we expressed 132 human IgG1 recombinant monoclonal antibodies (mAbs) using cDNA sequences of single Pfs230D1-specific memory B cells. The gating strategy is shown in Supplementary Fig. 1. We then tested their capacity for binding Pfs230D1 (by ELISA) and for functional activity (Fig. 1a). PBMCs from the different groups (AS01 vs Alhydrogel) were selected from participants presenting comparable antibody titers and serum functional activity in the different trials (Supplementary Fig. 2). Pre-vaccination levels of anti-Pfs230D1 are minimal and infrequent in malaria-exposed individuals in Mali (Supplementary Fig. 3). Thus, the anti-Pfs230D1 responses seen here are considered vaccine-driven. The function of antibodies was reported as TRA (Transmission Reducing Acti*vity)*, measured as the capacity of the mAb to reduce the parasite (oocyst) burden in mosquitoes a week after feeding on infective blood^16,17^, an assay referred to as standard membrane feeding assay (SMFA). Functional mAbs were defined here as those that achieved TRA > 75% (reduced number of oocysts by ≥75% versus control) at 100 µg/mL in the infective bloodmeal.

The binding epitopes of selected functional antibodies were recently reported^18^. The panel comprised 35 mAbs derived from Alhydrogel® recipients and 97 from AS01 recipients. Sequences were selected for mAb expression based on evidence of clonal expansion, as previously reported^16^. The frequency of mAbs binding Pfs230D1 (domain 1 of Pfs230 protein) in ELISAs was substantially higher in the Alhydrogel® group (32/35, 91.4%) than in the AS01 group (33/97, 34.0%) (Fig. 1b, c).

The overall percentages of functional antibodies were similar, 11.4% for Alhydrogel and 8.2% for AS01 (Fig. 1b, c). However, when analyzing the number of functional mAbs among Pfs230D1-binding antibodies, this number was higher for AS01 than for Alhydrogel®: 8 out of 33 (24.2%) AS01 Pfs230D1-binding mAbs were functional versus only 4 out of 32 (12.5%) from the Alhydrogel® group.

Interestingly, while all mAbs from the Alhydrogel® group using IGHV1-18 (14/14) or IGHV1-69 (10/10) in their heavy chain bound successfully to recombinant Pfs230D1 (Fig. 1d), none of these were functional mAbs (Fig. 1e). Sequences of the 97 mAbs from the AS01 group included 33 Pfs230D1-binding antibodies that were distributed among 14 different V genes in the IGHV1, IGHV3, and IGHV4 gene families (Fig. 1f). Similar to Alhydrogel® recipients, IGHV1-18 and IGHV1-69 genes were most frequent among AS01 recipients, being used by 18 and 30% of binding antibodies, respectively (Fig. 1f); 2/6 Pfs230D1-binding IGHV1-18 and 0/10 IGHV1-69 from AS01 group were functional (Fig. 1g). Among the sequences of 8 functional mAbs obtained from AS01 group, 2 were from IGHV1 gene family, 4 from IGHV3, and 2 from IGHV4 (Fig. 1f). Notably, both Alhydrogel® and AS01 formulations generated functional mAbs using IGHV3-21 and IGHV3-30 genes (Fig. 1e, g).

Thirteen binding mAbs from the Alhydrogel group used IGHD6-13 genes in their sequence (Supplementary Fig. 4). Interestingly, 12/14 (85.7%) of the mAbs using IGHV1-18 also used IGHD6-13 genes (which represented 12/13 (92.3%) mAbs containing IGHD6-13 genes). Despite their ability to bind to recombinant antigen, none of the mAbs containing IGHD6-13 genes was functional (Supplementary Fig. 4). mAbs derived from AS01 recipients used diverse D genes and no specific gene appeared to be more frequent among the binding or functional antibodies (Supplementary Fig. 4).

For the Alhydrogel® group, the majority of Pfs230D1-binding antibodies used IGHJ3 and IGHJ4 genes, while the few functional antibodies used IGHJ2 (2 mAbs) and IGHJ4 (2 mAbs) (Supplementary Fig. 5). For AS01 group, IGHJ4 was the most frequently used J gene family among Pfs230D1-binding and functional antibodies (19/33), followed by IGHJ5 and IGHJ6 (Supplementary Fig. 5).

### Association between Pfs230D1 human mAbs binding and CDR3 length

**Fig. 2:**
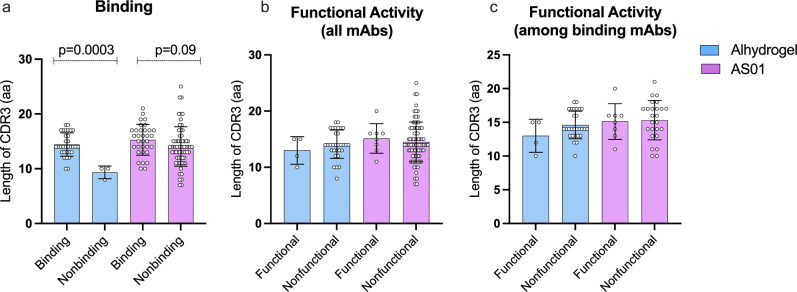
Association between heavy chain CDR3 length and binding or function of Pfs230D1 mAbs. **a** Binding was assessed by ELISA and compared between binding vs. non-binding mAbs in the two trials. **b**, **c** Functional activity was determined by Standard Membrane Feeding Assay (SMFA) and compared between functional and non-functional mAbs among **b** all mAbs, or **c** only those mAbs that bound antigen. *P* values were considered significant when <0.05, and statistical analyses were performed using unpaired *t* tests. Means and standard deviation are shown.

Among mAbs from Alhydrogel® group, CDR3 length (measured as the number of amino acids, aa) was higher for binding mAbs compared to non-binding mAbs (*p* < 0.005) (14 and 9 aa, respectively) (Fig. 2a). The longest CDR3 sequence of mAbs from Alhydrogel® group was identified in a binding antibody (18 aa) while the shortest was in a non-binding antibody (8 aa). For AS01 group, binding and non-binding mAbs had an average CDR3 length of 15 and 14 aa, respectively (*p* = 0.09); the longest CDR3 was found in binding mAbs (21 aa) and the shortest in non-binding (7 aa).

No association was determined between functional activity and CDR3 length (Fig. 2b). Average CDR3 length for functional and non-functional mAbs was 13 aa and 14 aa, respectively for Alhydrogel® group, versus 15 aa and 15 aa for AS01 group (Fig. 2b). Similar results were obtained after comparing functional vs. non-functional antibodies among only the mAbs known to bind antigen (Fig. 2c).

## Discussion

Malaria TBVs aim to reduce parasite burden in mosquitoes carrying the pathogen *Plasmodium*, thus reducing malaria transmission^19,20^. The leading TBV target antigen is Pfs230 (a 230 kDa protein present on the surface of *P. falciparum* gametes). Vaccines containing domain 1 of Pfs230 (Pfs230D1) adjuvanted with Alhydrogel® or AS01 are currently under evaluation in field trials in malaria-endemic areas^21^, (NCT05135273, NCT03917654), after a recent successful clinical trial in malaria-naive subjects^22^. To achieve a highly functional vaccine, antibodies targeting Pfs230 must be potent enough to block the transmission of parasites to mosquitoes. Understanding whether and how formulation with different adjuvants shapes the human antibody repertoire in response to Pfs230D1 is important to define B-cell immunity to TBVs. Antibody repertoire analyses by B cell sequencing have been useful to determine common signatures in antibody genes and gain insights into the adaptive immune response to other vaccines^23^. Here, we investigated the Pfs230D1 antibody repertoire at the antigen-specific, single-cell level, in response to formulations with Alhydrogel^®^ or AS01 (Pfs230D1-EPA/Alhydrogel^®^ and Pfs230D1-EPA/AS01).

Adjuvants can alter the selection of T-cell receptor (TCR) responses, activate human B cells through Toll-like receptor (TLR) ligands^24^, and increase the number of antigen-specific sequences in the B-cell receptor (BCR) repertoire^25^. In a mouse model of malaria vaccination, antibody repertoire sequencing determined that a TLR agonist adjuvant increased antibody variability in the animals and improved antibody binding to heterologous *Plasmodium* parasite strains^26^. AS01 adjuvant can promote robust immune responses during vaccination with CSP, the target antigen in the recently licensed *P. falciparum* pre-erythrocytic vaccine RTS,S; vaccination with CSP virus-like particles formulated in AS01 (RTS,S/AS01E vaccine) induced high anti-CSP serum levels in malaria-naive subjects^27^ and conferred protection against clinical malaria in young African children^28^.

Recent data have shown that a delayed, fractional 3rd dose of RTS,S/AS01 can increase protection against controlled human malaria infection from 62.7% (0–1–2 month regimen) to 87.5% (0–1–7 month regimen)^29^. In a study of over 100 mAbs obtained from plasmablasts of subjects receiving full or fractional RTS,S/AS01 regimens, IGHV3 heavy chain genes were the most frequent in response to RTS,S/AS01, especially IGHV3-30 and IGHV3-33^30^. Furthermore, IGHV3 genes have been identified in functional mAbs isolated after repeated immunization of malaria-naive volunteers with infectious *P. falciparum* sporozoites (PfSPZ) under chloroquine prophylaxis (PfSPZ-CVac)^31^. While our results showed that 4 out of the 8 functional mAbs from the AS01 group belonged to IGHV3, any correlation between the IGHV3 and functional activity for Pfs230D1-EPA/AS01 or RTS,S/AS01 vaccines remains to be investigated, including any adjuvant-specific effect. IGHV3 genes also predominate in anti-CSP responses after direct venous inoculation of PfSPZ (whole organism vaccine that does not incorporate additional adjuvant)^32,33^ or after naturally acquired infection^34^.

Although most IGHV1-69 antibodies in this study bound to recombinant Pfs230D1 in ELISA, none of them blocked parasite transmission when tested by SMFA. Although V gene usage by itself cannot predict whether an antibody will bind or not, IGHV1-69 genes are highly associated with cross-reactive binding in response to influenza vaccine^35,36^. Another study showed that the IGHV1-69 gene is three times more abundant in mutated clusters compared to unmutated clusters. In fact, IGHV1-69 is one of the most polymorphic genes within the human IGHV gene cluster^37,38^. IGHV1-69 polymorphisms seem to be substantially increased in African compared to Asian populations, for example^39^, and our study was conducted among Malian subjects in west Africa. Curiously, IGHV1-69 was used by LMIV230-01, a functional mAb isolated from a Malian adult enrolled in the same Alhydrogel trial as the subjects from the present study^17^. Thus, we cannot exclude the fact that IGHV1-69 might be increased in clonally expanded cells and eventually generate functional sequences.

In our study CDR3 length was significantly longer in mAbs isolated from the Alhydrogel^®^ cohort whose sequences bound to recombinant Pfs230D1 compared to those that did not bind. CDR3 length, however, was not associated with functional activity for any of the groups tested. Although the frequency of somatic hypermutation was increased in the Alhydrogel group compared to AS01 (Supplementary Fig. 6), we did not find differences in the CDR3 length for binding mAbs in either group. Our data suggest that AS01 generates a higher diversity of sequences accompanied by fewer clonal expansions (Supplementary Fig. 7). A recent study has demonstrated that the number of mutations in heavy chain genes does not correlate with CDR3 length^40^. In that study, antibody repertoire analyses in response to COVID-19 vaccines revealed that the average number of nucleotide mutations in IGH was 4.2 after the first dose and 11.7 after to the second dose, while the CDR3 length of IGH was unaltered after both doses^40^. We did not find a convergence between the CDR3s of mAbs isolated from the different adjuvant groups. However, we found a convergence of CDR3 within mAbs from the Alhydrogel group (Supplementary Fig. 8). Overall, we conclude that the Alhydrogel® adjuvanted malaria vaccine induced a more clonally expanded repertoire with higher SHM and longer CDR3s, compared to AS01 adjuvanted vaccine which induced a more diverse repertoire.

Our study has limitations. Time points of collection differed: Alhydrogel^®^ group, 14 days after dose 4; AS01 group, 7 days after dose 3. This was unavoidable for the AS01 group: at the time of this study, post-dose 4 samples were not yet available. However, this limitation was addressed in part by selecting samples with comparable antibody titers and serum functional activity between groups from the different trials (Supplementary Fig. 1). The second limitation was that a binomial readout (i.e., either TRA was higher or lower than 75% at 100 μg/mL) was used to judge the functionality of mAbs due to the low throughput of SMFA. A further study is required to assess the correlation between the antibody gene repertoire and quantitative functional activity (e.g., the concentration of each mAb, which gives 80% inhibition in TRA).

This work provides novel information on the diversity of antibody clonotypes elicited by vaccination with malaria TBVs and potential relationships between the use of adjuvants and V gene usage, CDR3 length, binding to recombinant protein, and functional blocking activity. Further research is required to explain why highly mutated IGHV1-69 sequences in this study bind to recombinant Pfs230 but cannot access the epitope on the surface of the parasites.

## Methods

### Human ethics statement

The clinical trials were approved by the FDA, by the ethics review boards from the Faculté de Médecine de Pharmacie et d’OdontoStomatologie (FMPOS), Bamako, Mali, and the US National Institute of Allergy and Infectious Diseases (NIH, Bethesda, MD, USA), as well as the Mali national regulatory authority. Written informed consent was obtained from study participants. The clinical trials are registered in clinicaltrials.gov (NCT02334462 for Pfs230D1-EPA/Alhydrogel, with results published^21^; NCT02942277 for Pfs230D1-EPA/AS01) and phase I in US healthy adults was published elsewhere^22^.

### Human immunization and samples collection

Malian adults were vaccinated with 40 µg of Pfs230D1-EPA in Alhydrogel^®^ or in AS01 at planned study days 0, 28, 168, and (for Alhydrogel^®^ group) 540. Pfs230D1-EPA/Alhydrogel^®^ 3rd and 4th doses were administered just before the rainy season (period of peak malaria transmission) in Year 1 and Year 2, respectively, of the study. From the Pfs230D1-EPA/Alhydrogel^®^ trial, sera and peripheral blood mononuclear cells (PBMCs) were obtained from eight participants, 14 days after the 4th dose (day 554). From the Pfs230D1-EPA/AS01 trial, collections occurred 7 days after the 3rd dose. PBMCs (5 million cells per sample on average) were prepared for isolation of Pfs230D1-specific single memory B cells.

### ELISA

ELISA to assess total anti-Pfs230D1 IgG levels in sera from immunized subjects and evaluation of binding activity of human mAbs was performed as previously reported^17^. Briefly, Immulon® 4HBX plates were coated with 1ug/well of recombinant Pfs230D1. Plates were incubated overnight at 4 °C and blocked with 320 µL of buffer containing 5% skim milk powder in Tris-buffered saline for 2 h at room temperature. Plates were washed with Tween-TBS. Samples (dilution 1:500 of sera or 100 μg/mL of mAbs) were added to Pfs230D1-coated wells, in triplicate, and incubated for 2 h at RT. Plates were washed, then 100 µL of alkaline phosphatase labeled goat anti-human IgG were added. Plates were incubated for 2 h at room temperature and washed. A colorimetric substrate, p-nitrophenyl phosphate (Sigma, St. Louis, USA) was added and plates were read at absorbances of 450 nm and 550 nm on a multi-well reader (Molecular Devices, San Jose, USA).

### Identification and sorting of antigen-specific single memory B cells

Identification and sorting of Pfs230D1M-specific B cells was performed as previously described^17^. Briefly, recombinant Pfs230D1M was chemically biotinylated using EZ-Link Sulfo-NHS-LC-Biotin (Thermo Fisher Scientific, Waltham, USA). The biotinylated protein was then tetramerized with streptavidin labeled with Phycoerythrin (PE) (Prozyme, Hayward, USA). PBMCs from vaccinees were thawed in 37 °C water bath, and resuspended in complete Roswell Park Memorial Institute (RPMI) 1640 Medium with L-glutamine and 25 mM HEPES (Corning, Corning, NY, USA, 10-041-CV) and washed with phosphate-buffer solution (PBS) (Thermo Fisher Scientific, Waltham, MA, USA, 10010023). One µL of Pfs230D1 tetramers was added to the cells that were then incubated at 4 °C for 20 minutes. Cells were washed with PBS containing 10% fetal bovine serum (FBS) and incubated with 25 µL of anti-PE magnetic beads (Miltenyi Biotech, Bergisch Gladbach, Germany, 130-048-801) for 25 minutes. Four mL of PBS were added to the solution, which was then passed over magnetic LS columns for elution of cell suspension enriched for antigen-specific cells. After enriching with the tetramer, PBMCs were stained with the following surface-conjugated antibodies: CD3 (UCHT1), CD14 (M5E2), CD56 (HCD56) Alexa Fluor 700, CD19 APC-CY7 (HIB19), CD20 PE-CY7 (2H7) and CD27 APC (LG.3A10), purchased from Biolegend (San Diego, USA). The following gating strategy was used: singlet cells were selected for the exclusion of non-B cells using CD3, CD14 and CD56 markers. Lymphocytes were gated for CD19+ CD20+. Pfs230D1M-specific B cells were gated using PE and excluding non-Pfs230D1M cells gated using CF594, the fluorochrome used in the decoy BSA tetramer. Sorting was performed into 96-well plates using a FACSAria™ II instrument (BD Biosciences, San Jose, USA) with blue, red, and violet lasers. Pfs230D1M-specific memory B cells were analyzed according to the fluorescence staining profile described above and sorted directly into 96-well PCR plates using a 100 µM nozzle. After sorting, plates were immediately centrifuged at 1,278 x g for 30 seconds, transported in dry ice and stored at −80 °C.

### Sequencing and data processing

Amplification of heavy and light chains and sequencing was performed by iRepertoire Inc. (Huntsville, AL, USA) as previously reported^17^. Briefly, amplification of BCR heavy and light chains from single sorted cells was performed by iRepertoire Inc. (Huntsville, AL, USA). RT-PCR1 was performed with nested, multiplex primers covering both heavy, kappa, and lambda loci, and including partial Illumina adaptors. Included on the reverse primer was an in-line six nucleotides barcode, which served as a plate identifier so that multiple 96-well plates could be multiplexed in the same sequencing flow cell. After RT-PCR1, the first round PCR1 products were rescued using SPRISelect Beads (Beckman Coulter, Brea, USA). A second PCR was performed with dual-indexed primers that complete the sequencing adaptors introduced during PCR1 and provide plate positional information for the sequenced products. Sequencing was performed using the Illumina MiSeq v2 500-cycle kit with 250 paired-end reads.

### Standard membrane feeding assay to assess functional activity of polyclonal antibodies in serum of vaccinees or Pfs230D1 mAbs

Transmission reducing activity (TRA) was measured as the reduction of mean *P. falciparum* burden in infected mosquitoes fed with intact sera from vaccinees, using the SMFA, as previously reported^22^. Percentage reduction of *P. falciparum* oocyst numbers was calculated for immune serum as compared to negative control (naive sera from US donors). Additional negative control (mouse anti *P. yoelii* P140 at 375 µg/mL) was used to confirm the oocyst reduction activity of the Pfs230D1 mAbs (human IgG1 isotype at 1000 µg/mL in pooled sera from US malaria-naive donors). A mouse anti-Pfs25 mAb (4B7, at 175 µg/mL) was used as a positive control.

Briefly, an in vitro 15-day culture of *P. falciparum* (NF54 line) containing stage V gametocytes was diluted with washed O + RBCs (Interstate Blood Bank, Memphis, USA) and an AB + serum pool (not heat-inactivated) from US malaria-naive subjects (Interstate Blood Bank, Memphis, USA) to final concentration of 0.07%–0.1% stage V gametocytes and 50% hematocrit. For each individual assay, 200 µL of diluted culture was mixed with 60 µL of mAb diluted in PBS. Samples were then fed to pre-starved (24–30 h) 3–8-day-old Anopheles stephensi (Nijmegen strain) mosquitoes using a Parafilm membrane on a mosquito feeder, kept warm with 40 °C circulating water. After feeding, mosquitoes were kept at 26 °C and 80% humidity conditions to allow parasites to develop. On Day 8 after the feed, mosquito midguts were dissected and stained with 0.05–0.1% mercurochrome solution in water for 20–30 min. Infectivity was measured by counting oocysts in at least 20 mosquitoes per sample. Each sample was tested in at least two independent SMFAs. For SMFA experiments performed to assess the complement-dependent activity of the mAbs, sera were heat-inactivated at 56 °C before the addition of mAbs.

### BCR sequence analyses

2393 sequences were assigned Ig genes, out of 2,397 input sequences (99.83%). Gene assignment and other downstream analysis were performed with the Immcantation suite container version 4.1.0, which includes IMGT reference germlines downloaded on 2020-08-12, IgBLAST version 1.16.0, Change-O 1.0.0, Alakazam 1.0.2 and SHazaM 1.0.2. After gene assignment with IgBLAST, 126 unproductive sequences were removed. Data were processed to retain one heavy chain sequence and, at most, two light chain sequences per well. In wells with multiple heavy chain sequences, we retained the one with the highest cdr3_read_count, and required it to account for more than 50% of the total heavy chain cdr3_read_count in the well. In wells with more than two light chain sequences, we retained the two sequences with the highest cdr3_read_count, each accounting for more than 25% of the total light chain cdr3_read_count in the well, and together for >75% of the total light chain cdr3_read_count in the well. Sequences from wells presenting light chain only were removed from the analysis. One well was excluded for possible contamination (subject 2, well A07, Pfs230 Alhydrogel). A total of 1,345 sequences passed these filters (733 heavy, 612 light), and constitute the final set of sequences analyzed (Supplementary Table 1), with an average of 57 sequences per subject for the Alhydrogel group, and 60 sequences for the AS01 group. Most of the sequences (79.26%) from the heavy chain sequences correspond to VH:VL pairs (Supplementary Fig. 4). Most of these analyses consisted in descriptive data and statistical comparison were not performed for all figures.

### Sequence selection, mAbs expression, and purification

VH and VL sequences of Pfs230D1-specific single memory B cells were selected for expression based on evidence of clonal expansion. If multiple cells contained identical CDR3 sequences, it was attributed to clonal expansion, and thus evidence of activation. Naturally paired VH/VL sequences were expressed in an IgG1 backbone by LakePharma Inc. Monoclonal antibodies were cloned based on the criteria above, and VH/VL pairs for cloning and expression were obtained from all participants in this study.

Human mAbs were expressed using 0.1 L transient production in HEK293 cells (Thermo Fisher Scientific) and purified using protein A affinity chromatography. Purified proteins were sterilized using a 0.2 μm sterile filter and characterized to confirm 90% purity by capillary electrophoresis-SDS, concentration, and endotoxin levels according to the manufacturer’s procedures.

### SMFA to test functional activity of mAbs

SMFA was performed to assess the ability of mAbs to block the development of *P. falciparum* strain NF54 oocysts in the mosquito midgut, as previously reported^41^. Each mAb was evaluated once at each test concentration, and *n* = 20 mosquitoes were examined per sample. In each assay, as a control, normal mouse antibody at 750 µg/mL was included (*n* = 20 or 40 mosquitoes).

All the mAbs from the AS01 group were tested at 100 µg/mL. The mAbs from the Alhydrogel group were initially screened at 375 µg/mL, and then the mAbs with >65% TRA at 375 µg/mL were further tested at 100 µg/mL. A functional mAb was defined as a mAb with >75% TRA at 100 µg/mL. If a mAb showed <65% TRA at 375 µg/mL (it was not tested at 100 µg/mL by SMFA), the mAb was categorized as “non-functional”.

### Statistical analyses

Statistical analyses of antibody genes were performed using Wilcoxon rank sum test. The Benjamini–Hochberg false discovery rate (FDR) was used to correct for multiple hypothesis testing, and the criteria for significance were *p* < 0.05 and FDR < 0.20. The descriptions of the tests used are contained within the figure legends.

### Supplementary information

Supplementary Material

Supplementary Data 1

Supplementary Data 2

## Data Availability

Data are provided in the Supplementary material.

## Code availability

Computer code used for analysis can be made available upon reasonable request to the authors.
