# Supplementary material for: Antibody gene features associated with binding and functional activity in malaria vaccine-derived human mAbs
Source: NPJ Vaccines. 2024 Aug 10;9:144. doi: 10.1038/s41541-024-00929-6 (PMC11316794; doi:10.1038/s41541-024-00929-6)
Supplement: Supplementary file 1 — Supplementary Material [file 41541_2024_929_MOESM1_ESM.pdf]

**SUPPLEMENTARY MATERIAL**

# Antibody gene features associated with binding and functional activity in malaria vaccine-derived human mAbs

Camila H. Coelho, Susanna Marquez, Bergeline C. Nguemwo Tentokam, Anne D. Berhe[1], Kazutoyo Miura, Vishal Rao, Carole A. Long, Ogobara K. Doumbo[4], Issaka Sagara, Sara Healy, Steven H. Kleinstein, Patrick E. Duffy

**Contents:**

Supplementary Figures 1-8

Supplementary Table 1

CONSORT Checklist for Pfs230D1-EPA/Alhydrogel in Mali trial

CONSORT Checklist for Pfs230D1-EPA/AS01 in Mali trial

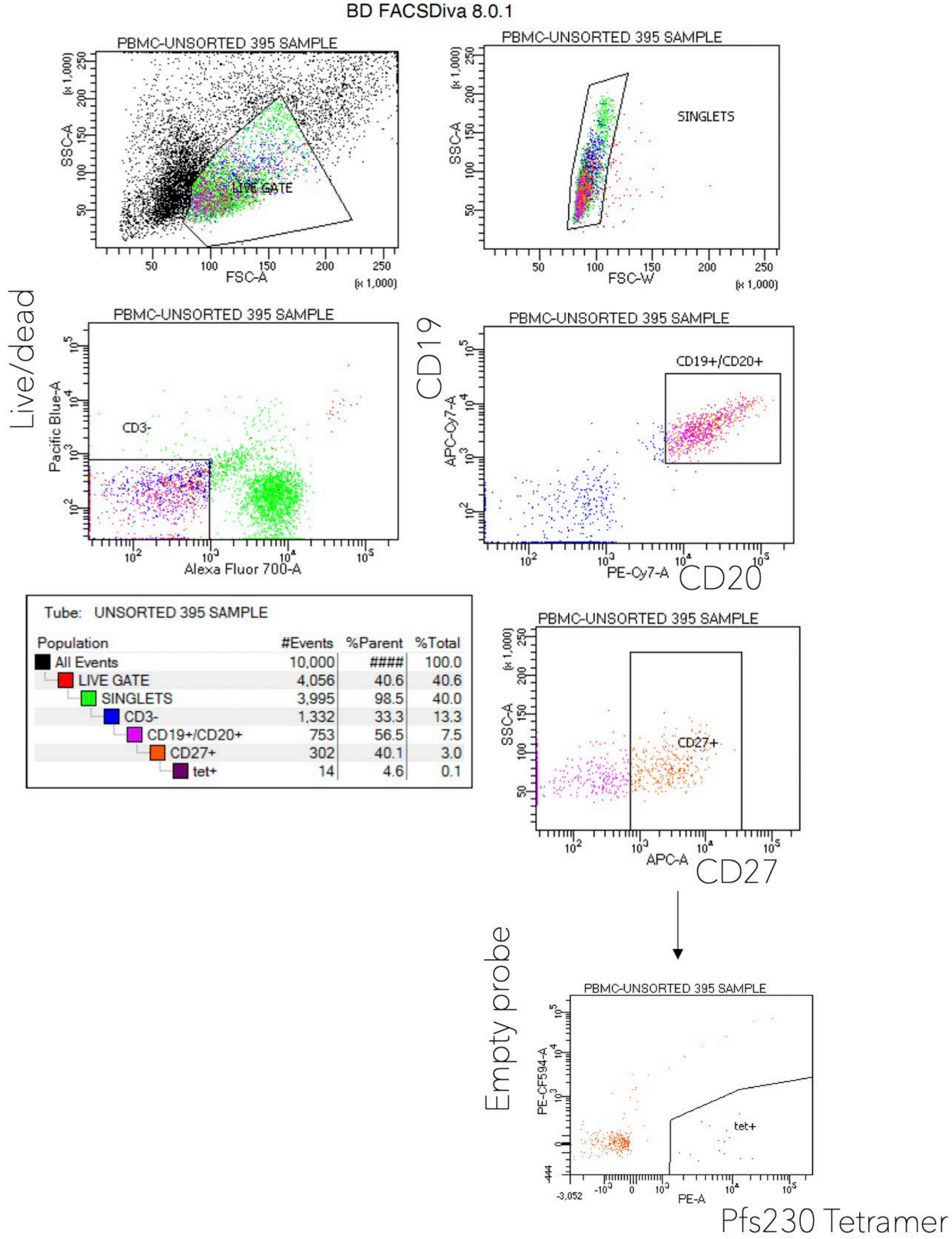


Tube: UNSORTED 395 SAMPLE

| Population | #Events | %Parent | %Total |
|---|---|---|---|
| All Events | 10,000 | #### | 100.0 |
| LIVE GATE | 4,056 | 40.6 | 40.6 |
| SINGLETS | 3,995 | 98.5 | 40.0 |
| CD3- | 1,332 | 33.3 | 13.3 |
| CD19+/CD20+ | 753 | 56.5 | 7.5 |
| CD27+ | 302 | 40.1 | 3.0 |
| tet+ | 14 | 4.6 | 0.1 |

**Supplementary Figure 1. Gating strategy to identify and sort Pfs230D1-specific single memory B cells by flow cytometry.** Tet = Pfs230D1 B cell tetramer.

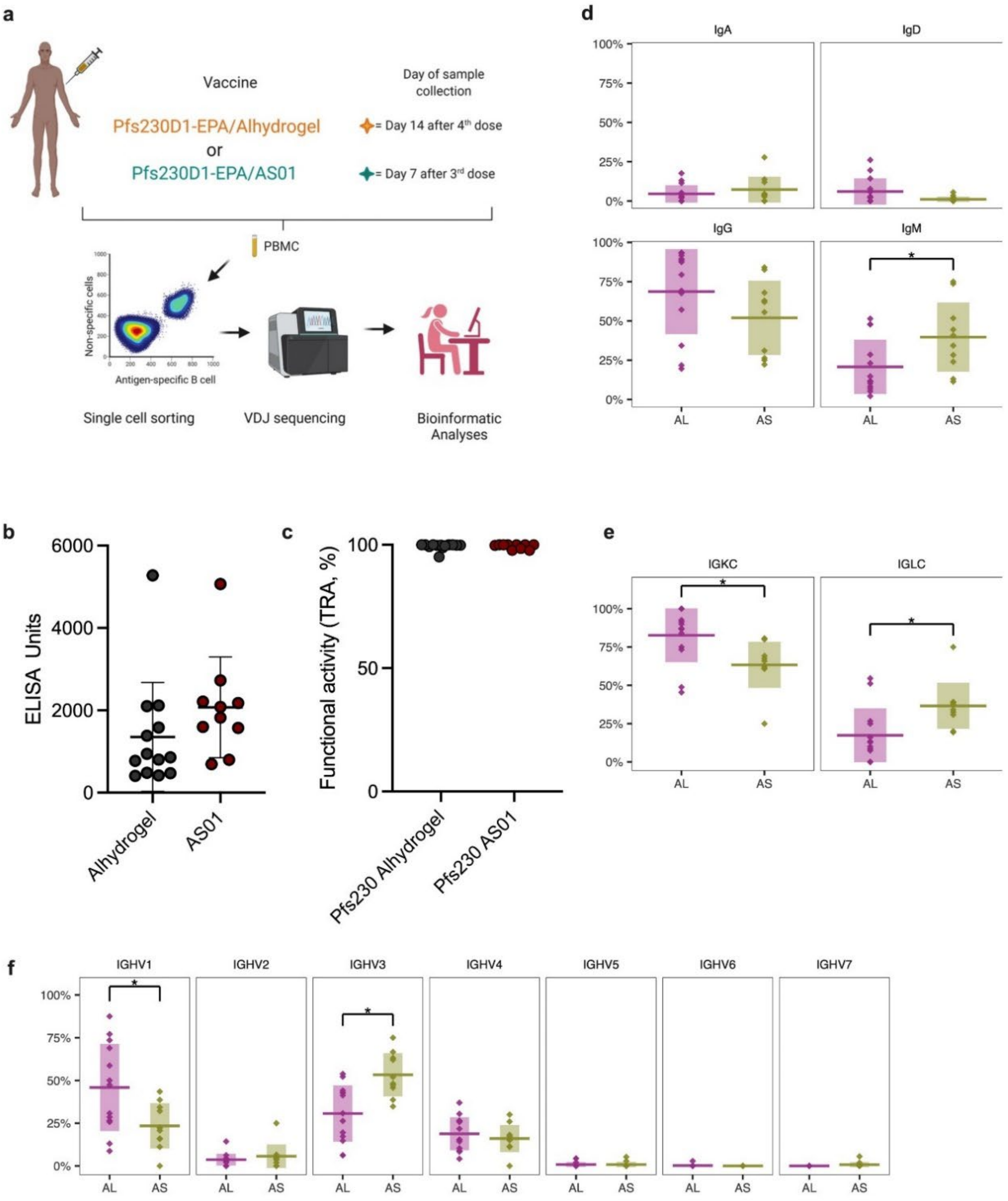


**Supplementary Figure 2. Antibody repertoire in Pfs230D1-specific single B cells in response to vaccination with Alhydrogel or AS01 adjuvants. (a)** Malian adults received 3 or 4 doses of Pfs230 conjugated with the carrier Exoprotein A and formulated with either Alhydrogel® or AS01 adjuvants. PMBCs were collected from subjects receiving Pfs230D1-EPA/Alhydrogel® (Pfs230AL) or Pfs230D1-EPA/AS01 (Pfs230 AS) and Pfs230D1-specific single B cells were sorted and had their B cell receptor sequenced. Bioinformatic analyses were performed using the Immcantation framework. Samples from the subjects enrolled in the clinical trial with vaccines formulated with Alhydrogel® were collected 14 days after the 4$^{th}$ dose, and with AS01, were obtained 7 days after the 3$^{rd}$ dose **(b)** Anti-Pfs230D1 IgG titers in response to both vaccines were measured by ELISA. **(c)** Serum functional activity was assessed by SMFA and determined by the ability to reduce the number of oocysts in midguts of infected *Anopheles* mosquitoes fed with *NF54 Plasmodium falciparum*. **(d)** Frequency of occurrence of isotypes IgA, IgD, IgG and IgM in the repertoire (in percentage) was compared between the Alhydrogel® (AL) and AS01 (AS) adjuvant groups. **(e)** Frequency of occurrence of the kappa (IGKC) and lambda (IGLC) light chains (in percentage) was compared between the Alhydrogel® and AS01 adjuvant groups. **(f)** Usage of heavy chain V-gene families was compared between the Alhydrogel® and AS01 adjuvant groups.

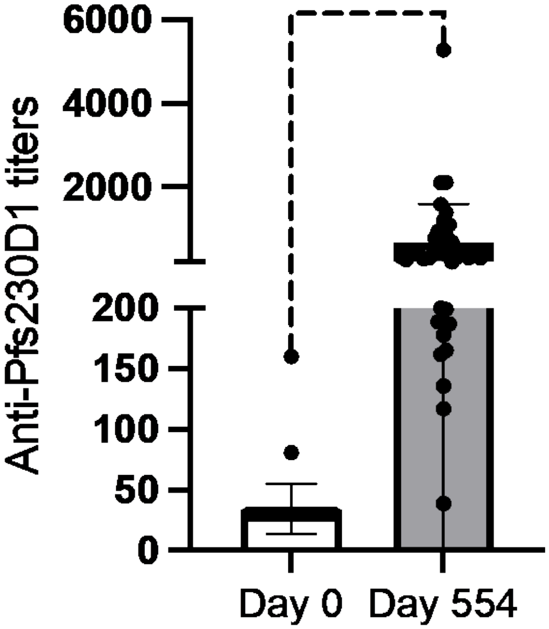


**Supplementary Figure 3. Anti-Pfs230D1 titers pre-vaccination and post-dose 4 (Alhydrogel) in Malian adults.** Binding was assessed by ELISA. Antibody reactivity below the level of quantification (LOQ) was assigned a value equal to the LOQ determined for that ELISA plate (30-40 ELISA units).

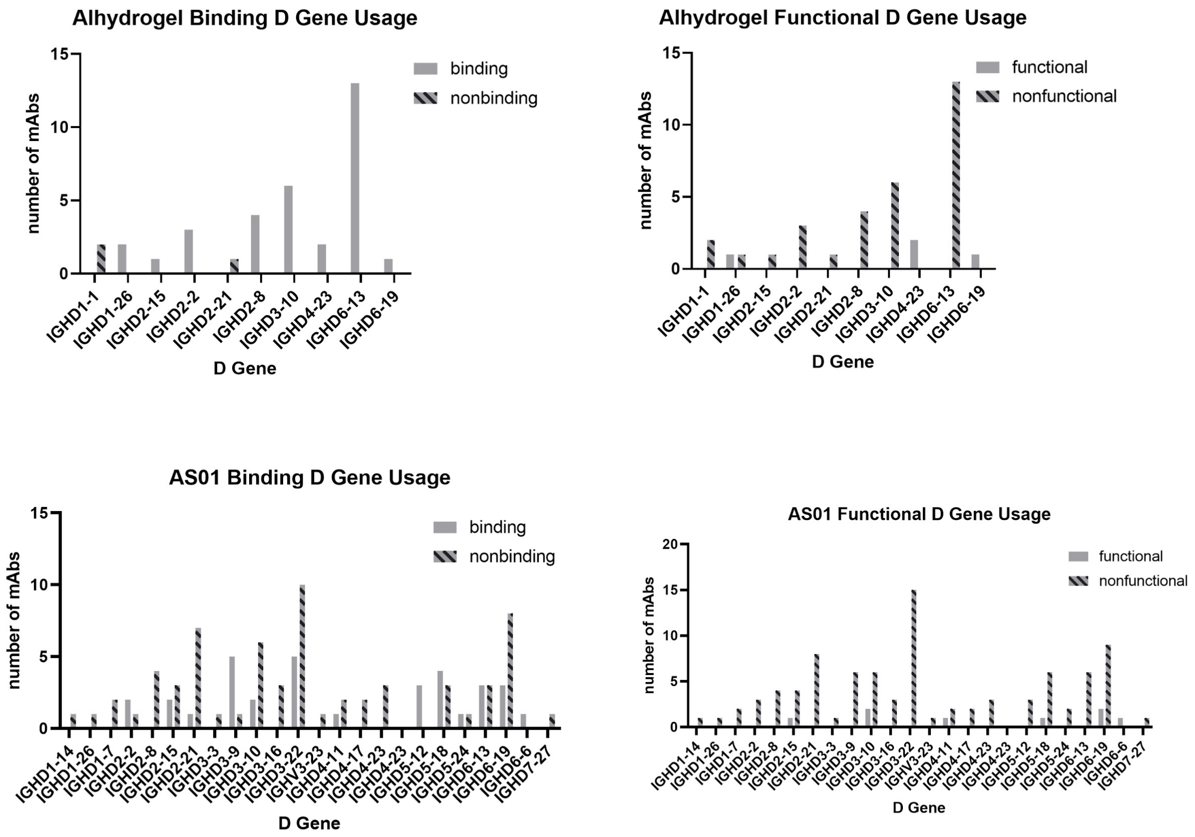


**Supplementary Figure 4. D genes present in mAbs, grouped by binding and functional activity profile.** Binding was assessed by ELISA and functional activity by SMFA. Functional antibodies are reported with functional activity higher than 75% at 100μg/mL.

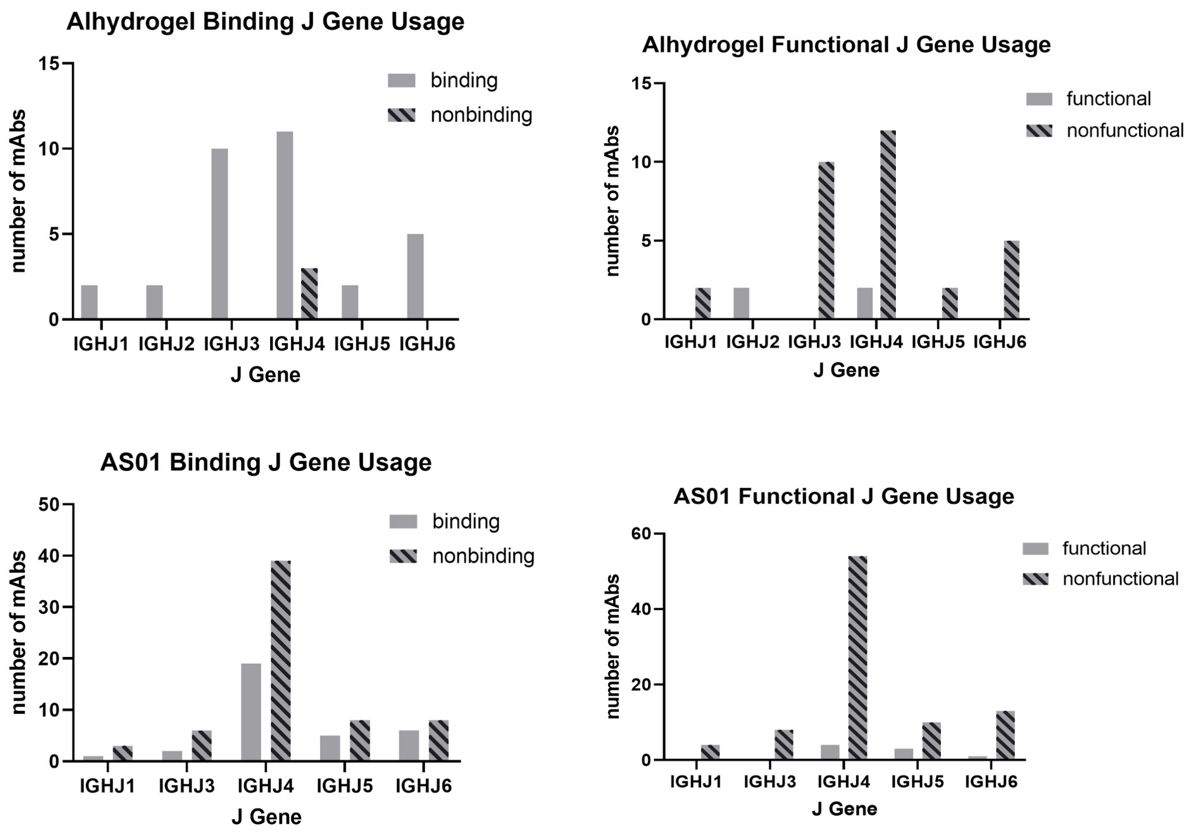


**Supplementary Figure 5. Heavy chain J genes present in mAbs, grouped by binding and functional activity profile.** Binding was assessed by ELISA, and functional activity was assessed by SMFA. Functional antibodies are reported as having functional activity higher than 75% at 100μg/mL.

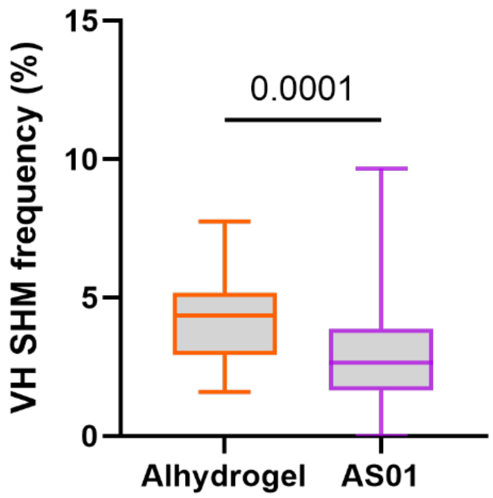


**Supplementary Figure 6. Somatic hypermutation levels in the sequences of the mAbs obtained from each group.**

## Alhydrogel

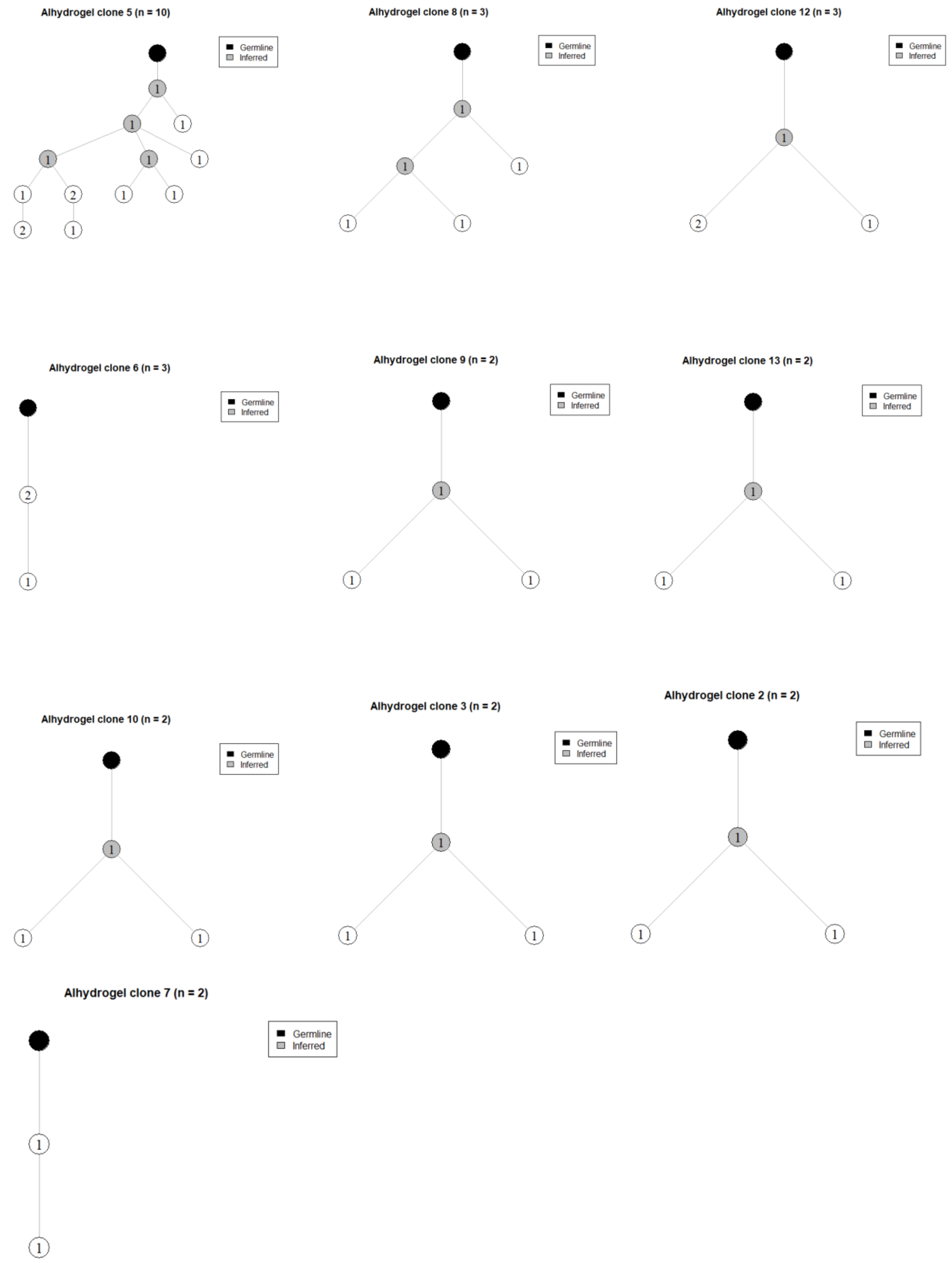


**Supplementary Figure 7.** Clonal expansions in the sequences of human monoclonal antibodies expressed from each of the adjuvant groups.

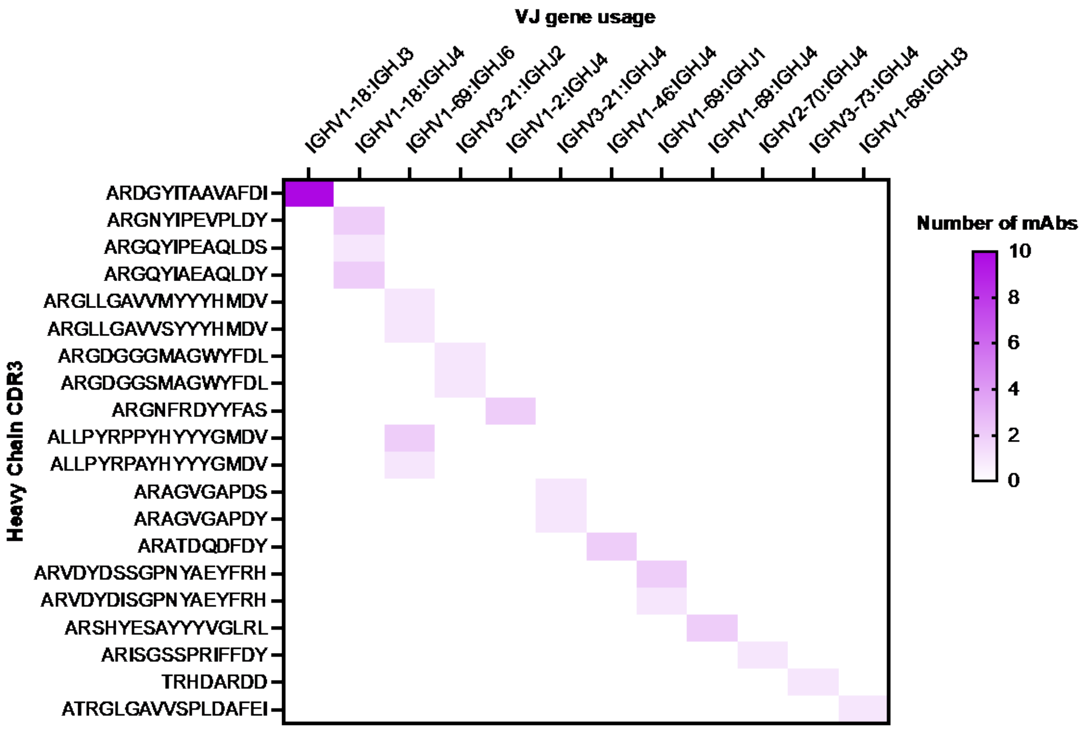


**Supplementary Figure 8.** Converge (identical aminoacid sequences) between CDR3 sequences within the Alhydrogel group. No convergence was determined between the two adjuvant groups.

| Alhydrogel (Subject ID) | Initial number sequences | Number of sequences after filtering | AS01 (Subject ID) | Initial number of sequences | Number of sequences after filtering |
|---|---|---|---|---|---|
| **1** | *69* | *34* | **1** | *69* | *50* |
| **2** | *183* | *96* | **2** | *55* | *35* |
| **3** | *112* | *54* | **3** | *16* | *8* |
| **4** | *148* | *87* | **4** | *152* | *94* |
| **5** | *98* | *46* | **5** | *123* | *70* |
| **6** | *71* | *22* | **6** | *119* | *94* |
| **7** | *106* | *58* | **7** | *78* | *52* |
| **8** | *154* | *81* | **8** | *165* | *89* |
| **9** | *82* | *45* | **9** | *109* | *75* |
| **10** | *125* | *61* | **10** | *52* | *31* |
| **11** | *37* | *9* | | | |
| **12** | *157* | *89* | | | |
| **13** | *117* | *65* | | | |
| TOTAL: | ***1459*** | ***747*** | TOTAL: | ***938*** | ***598*** |

**Supplementary Table 1 – Number of total BCR sequences per subject, including both VH and VL sequences.**

# CONSORT 2010 checklist of information to include when reporting a randomised trial*

## For Pfs230D1-EPA/Alhydrogel trial in Mali:

| Section/Topic | Item No | Checklist item | Reported on page No |
|---|---|---|---|
| **Title and abstract** | | | |
| | 1a | Identification as a randomised trial in the title | N/A |
| | 1b | Structured summary of trial design, methods, results, and conclusions (for specific guidance see CONSORT for abstracts) | N/A |
| **Introduction** | | | |
| Background and objectives | 2a | Scientific background and explanation of rationale | Ref 21: pp.1267-1268 |
| | 2b | Specific objectives or hypotheses | Ref 21: pp.1267-1268 |
| **Methods** | | | |
| Trial design | 3a | Description of trial design (such as parallel, factorial) including allocation ratio | Ref 21: pp.1268-1270 |
| | 3b | Important changes to methods after trial commencement (such as eligibility criteria), with reasons | Ref 21: Supp Appendix (SA) p. 31 |
| Participants | 4a | Eligibility criteria for participants | Ref 21: SA p. 9-10 |
| | 4b | Settings and locations where the data were collected | Ref 21: pp1267-1268 |
| Interventions | 5 | The interventions for each group with sufficient details to allow replication, including how and when they were actually administered | Ref 21: pp1268-1270 |
| Outcomes | 6a | Completely defined pre-specified primary and secondary outcome measures, including how and when they were assessed | Ref 21: 1270 |
| | 6b | Any changes to trial outcomes after the trial commenced, with reasons | Ref 21: SA p. 31 |

| | | | |
|---|---|---|---|
| Sample size | 7a | How sample size was determined | Ref 21: SA p.33 |
| | 7b | When applicable, explanation of any interim analyses and stopping guidelines | n/a |
| Randomisation: | | | |
| Sequence generation | 8a | Method used to generate the random allocation sequence | Ref 21: p1268 and SA p.7 |
| | 8b | Type of randomisation; details of any restriction (such as blocking and block size) | Ref 21: p1268 and SA p.7 |
| Allocation concealment mechanism | 9 | Mechanism used to implement the random allocation sequence (such as sequentially numbered containers), describing any steps taken to conceal the sequence until interventions were assigned | Ref 21: p1268 and SA p.7 |
| Implementation | 10 | Who generated the random allocation sequence, who enrolled participants, and who assigned participants to interventions | Ref 21: p1268 and SA p.7 |
| Blinding | 11a | If done, who was blinded after assignment to interventions (for example, participants, care providers, those assessing outcomes) and how | Ref 21: pp1268-1270 and SA p.7 |
| | 11b | If relevant, description of the similarity of interventions | n/a |
| Statistical methods | 12a | Statistical methods used to compare groups for primary and secondary outcomes | Ref 21: p1270-1273 |
| | 12b | Methods for additional analyses, such as subgroup analyses and adjusted analyses | Ref 21: SA pp21-23 |
| **Results** | | | |
| Participant flow (a diagram is strongly recommended) | 13a | For each group, the numbers of participants who were randomly assigned, received intended treatment, and were analysed for the primary outcome | Ref 21: p1269 |
| | 13b | For each group, losses and exclusions after randomisation, together with reasons | Ref 21: p1269 |
| Recruitment | 14a | Dates defining the periods of recruitment and follow-up | Ref 21: p1268-1270 |
| | 14b | Why the trial ended or was stopped | n/a |
| Baseline data | 15 | A table showing baseline demographic and clinical characteristics for each group | Ref 21: p1271 |

| | | | |
|---|---|---|---|
| Numbers analysed | 16 | For each group, number of participants (denominator) included in each analysis and whether the analysis was by original assigned groups | Ref 21: p1273 |
| Outcomes and estimation | 17a | For each primary and secondary outcome, results for each group, and the estimated effect size and its precision (such as 95% confidence interval) | Ref21: p1273-1275 |
| | 17b | For binary outcomes, presentation of both absolute and relative effect sizes is recommended | Ref 21: p1273-1275 |
| Ancillary analyses | 18 | Results of any other analyses performed, including subgroup analyses and adjusted analyses, distinguishing pre-specified from exploratory | Ref 21: pp1275-1277 |
| Harms | 19 | All important harms or unintended effects in each group (for specific guidance see CONSORT for harms) | Ref 21: pp1272 |
| **Discussion** | | | |
| Limitations | 20 | Trial limitations, addressing sources of potential bias, imprecision, and, if relevant, multiplicity of analyses | Ref 21: pp1277-1278 |
| Generalisability | 21 | Generalisability (external validity, applicability) of the trial findings | Ref 21: pp1277-1278 |
| Interpretation | 22 | Interpretation consistent with results, balancing benefits and harms, and considering other relevant evidence | Ref 21: pp1277-1278 |
| **Other information** | | | |
| Registration | 23 | Registration number and name of trial registry | Clinicaltrials.gov NCT02334462 |
| Protocol | 24 | Where the full trial protocol can be accessed, if available | Upon request from corresponding author |
| Funding | 25 | Sources of funding and other support (such as supply of drugs), role of funders | Page 10 |

Citation: Schulz KF, Altman DG, Moher D, for the CONSORT Group. CONSORT 2010 Statement: updated guidelines for reporting parallel group randomised trials. BMC Medicine. 2010;8:18. © 2010 Schulz et al. This is an Open Access article distributed under the terms of the Creative Commons Attribution License (http://creativecommons.org/licenses/by/2.0), which permits unrestricted use, distribution, and reproduction in any medium, provided the original work is properly cited.

*We strongly recommend reading this statement in conjunction with the CONSORT 2010 Explanation and Elaboration for important clarifications on all the items. If relevant, we also recommend reading CONSORT extensions for cluster randomised trials, non-inferiority and equivalence trials, non-pharmacological treatments, herbal interventions, and pragmatic trials. Additional extensions are forthcoming: for those and for up-to-date references relevant to this checklist, see www.consort-statement.org.

# CONSORT 2010 checklist of information to include when reporting a randomised trial*

# For Pfs230D1-EPA/AS01 trial in Mali:

| Section/Topic | Item No | Checklist item | Reported on page No |
|---|---|---|---|
| **Title and abstract** | | | |
| | 1a | Identification as a randomised trial in the title | n/a |
| | 1b | Structured summary of trial design, methods, results, and conclusions (for specific guidance see CONSORT for abstracts) | n/a |
| **Introduction** | | | |
| Background and objectives | 2a | Scientific background and explanation of rationale | Page 3 |
| | 2b | Specific objectives or hypotheses | Page 3 |
| **Methods** | | | |
| Trial design | 3a | Description of trial design (such as parallel, factorial) including allocation ratio | Page 7 (NCT02942277) |
| | 3b | Important changes to methods after trial commencement (such as eligibility criteria), with reasons | n/a |
| Participants | 4a | Eligibility criteria for participants | Page 7 (NCT02942277) |
| | 4b | Settings and locations where the data were collected | Page 7 (NCT02942277) |
| Interventions | 5 | The interventions for each group with sufficient details to allow replication, including how and when they were actually administered | Page 7 (NCT02942277) |
| Outcomes | 6a | Completely defined pre-specified primary and secondary outcome measures, including how and when they were assessed | Page 7 (NCT02942277) |
| | 6b | Any changes to trial outcomes after the trial commenced, with reasons | n/a |
| Sample size | 7a | How sample size was determined | Page 7 |
| | 7b | When applicable, explanation of any interim analyses and stopping guidelines | n/a |

| | | | |
|---|---|---|---|
| Randomisation: | | | |
| Sequence generation | 8a | Method used to generate the random allocation sequence | Not yet reported |
| | 8b | Type of randomisation; details of any restriction (such as blocking and block size) | Page 7 (NCT02942277) |
| Allocation concealment mechanism | 9 | Mechanism used to implement the random allocation sequence (such as sequentially numbered containers), describing any steps taken to conceal the sequence until interventions were assigned | Not yet reported |
| Implementation | 10 | Who generated the random allocation sequence, who enrolled participants, and who assigned participants to interventions | Not yet reported |
| Blinding | 11a | If done, who was blinded after assignment to interventions (for example, participants, care providers, those assessing outcomes) and how | Not yet reported |
| | 11b | If relevant, description of the similarity of interventions | n/a |
| Statistical methods | 12a | Statistical methods used to compare groups for primary and secondary outcomes | Not yet reported |
| | 12b | Methods for additional analyses, such as subgroup analyses and adjusted analyses | Not yet reported |
| **Results** | | | |
| Participant flow (a diagram is strongly recommended) | 13a | For each group, the numbers of participants who were randomly assigned, received intended treatment, and were analysed for the primary outcome | Page 7 (NCT02942277) |
| | 13b | For each group, losses and exclusions after randomisation, together with reasons | Page 7 (NCT02942277) |
| Recruitment | 14a | Dates defining the periods of recruitment and follow-up | Page 7 (NCT02942277) |
| | 14b | Why the trial ended or was stopped | n/a |
| Baseline data | 15 | A table showing baseline demographic and clinical characteristics for each group | Page 7 (NCT02942277) |
| Numbers analysed | 16 | For each group, number of participants (denominator) included in each analysis and whether the analysis was by original assigned groups | Page 7 (NCT02942277) |

| | | | |
|---|---|---|---|
| Outcomes and estimation | 17a | For each primary and secondary outcome, results for each group, and the estimated effect size and its precision (such as 95% confidence interval) | Not yet reported |
| | 17b | For binary outcomes, presentation of both absolute and relative effect sizes is recommended | Not yet reported |
| Ancillary analyses | 18 | Results of any other analyses performed, including subgroup analyses and adjusted analyses, distinguishing pre-specified from exploratory | Not yet reported |
| Harms | 19 | All important harms or unintended effects in each group (for specific guidance see CONSORT for harms) | Page 7 (NCT02942277) |
| **Discussion** | | | |
| Limitations | 20 | Trial limitations, addressing sources of potential bias, imprecision, and, if relevant, multiplicity of analyses | Page 7 (NCT02942277) |
| Generalisability | 21 | Generalisability (external validity, applicability) of the trial findings | Not yet reported |
| Interpretation | 22 | Interpretation consistent with results, balancing benefits and harms, and considering other relevant evidence | Not yet reported |
| **Other information** | | | |
| Registration | 23 | Registration number and name of trial registry | Clinicaltrials.gov NCT02942277 |
| Protocol | 24 | Where the full trial protocol can be accessed, if available | Upon request from corresponding author |
| Funding | 25 | Sources of funding and other support (such as supply of drugs), role of funders | Page 10 |

Citation: Schulz KF, Altman DG, Moher D, for the CONSORT Group. CONSORT 2010 Statement: updated guidelines for reporting parallel group randomised trials. BMC Medicine. 2010;8:18.
© 2010 Schulz et al. This is an Open Access article distributed under the terms of the Creative Commons Attribution License (http://creativecommons.org/licenses/by/2.0), which permits unrestricted use, distribution, and reproduction in any medium, provided the original work is properly cited.

*We strongly recommend reading this statement in conjunction with the CONSORT 2010 Explanation and Elaboration for important clarifications on all the items. If relevant, we also recommend reading CONSORT extensions for cluster randomised trials, non-inferiority and equivalence trials, non-pharmacological treatments, herbal interventions, and pragmatic trials. Additional extensions are forthcoming: for those and for up-to-date references relevant to this checklist, see www.consort-statement.org.
